# Diabetes in patients with acromegaly treated with pegvisomant: observations from acrostudy

**DOI:** 10.1007/s12020-018-1792-0

**Published:** 2018-11-24

**Authors:** Thierry Brue, Anders Lindberg, Aart Jan van der Lely, Ann Charlotte Akerblad, Maria Koltowska-Häggström, Roy Gomez, Michael Droste, Judith Hey-Hadavi, Christian J Strasburger, Cecilia Camacho-Hübner

**Affiliations:** 1 Aix-Marseille Université, Institut National de la Santé et de la Recherche Médicale (INSERM), U1251, Marseille Medical Genetics (MMG), 13005 Marseille, France; 2 Assistance Publique-Hôpitaux de Marseille (AP-HM), Department of Endocrinology, Hôpital de la Conception, Centre de Référence des Maladies Rares de l’hypophyse HYPO, 13005 Marseille, France; 3 Pfizer Health AB, Sollentuna, Sweden; 4 Department of Medicine, Erasmus University MC, Rotterdam, The Netherlands; 5 Department of Women’s and Children’s Health, Uppsala University, Uppsala, Sweden; 6 European Medical Affairs, Pfizer, 1050 Brussels, Belgium; 7 Practice for Endocrinology and Diabetes, Oldenburg, Germany; 8 Endocrine Care, Pfizer Inc, New York, NY USA; 9 Division of Clinical Endocrinology, Department of Medicine, Campus Charité Mitte, Berlin, Germany

**Keywords:** Acromegaly, Diabetes, PEGV, HbA1c, IGF-I, Surveillance study

## Abstract

**Purpose:**

To explore the effects of pegvisomant (PEGV) on glucose metabolism in patients with acromegaly within ACROSTUDY, an international, observational, prospective safety surveillance study.

**Methods:**

Patients were retrospectively divided into two cohorts, with (DM group) or without diabetes mellitus (no-DM). Parameters of glucose metabolism and IGF-I values were analyzed yearly both cross-sectionally for 4 years (yrs) and longitudinally at 1 and 4–5 yrs of PEGV treatment.

**Results:**

Among 1762 patients, 510 (28.9%) had DM before PEGV start. At cross-sectional analyses, in the DM group mean blood glucose was 140.0 ± 58.7 mg/dl at baseline, 116.4 ± 44.8 mg/dl at year 1 and 120.0 ± 44.3 mg/dl at yr 4. Mean HbA1c was 6.6 ± 1.2 % at yr 1 vs. 7.0 ± 1.4 % at baseline. HbA1c was above 6.5% in 61.9% at baseline and ranged from 45.4 to 53.8% at subsequent yearly time points. At the 4-yr longitudinal analysis, in the DM group (*n* = 109), mean blood glucose decreased by 20.2 mg/dl at yr 4, mean HbA1c was 7.0 ± 1.5% at baseline vs. 6.8 ± 1.4%. Patients achieved IGF-I normalization in 52.1% and 57.4% of cases in the DM and no-DM groups, respectively at 1 year. The mean daily PEGV dose (mg/day) was higher in the DM group (18.2 vs. 15.3) while the absolute change of IGF-I values from baseline was similar in both groups. PEGV was well tolerated in both groups without any unexpected AEs.

**Conclusions:**

Patients with DM had a moderate decrease in mean fasting glucose values during PEGV treatment.

## Introduction

Growth hormone (GH) has anabolic effects, but also antagonizes insulin actions as it stimulates lipolysis, gluconeogenesis, and glycogenolysis [1, 2]. Acromegaly is almost always caused by pathological hypersecretion of GH by a somatotropinoma of the anterior pituitary [3, 4]. Thus, uncontrolled patients with acromegaly frequently have diabetes, or at least impaired glucose tolerance (IGT), as reported decades ago [5] and confirmed by more recent studies like the Liege Acromegaly Survey where 27.5% of patients had diabetes mellitus (DM) at the time of their diagnosis [6]. Medical treatment modalities for acromegaly may in turn reduce insulin resistance and increase insulin sensitivity [7]. These treatments include somatostatin analogues (SSAs), dopamine agonists, and the GH-receptor (GHR) antagonist pegvisomant (PEGV) [3]. In a meta-analysis, it was reported that classically used SSAs (octreotide and lanreotide) may have a marginal negative clinical impact on glucose homeostasis in acromegaly [8] while in a head-to-head study vs. octreotide, hyperglycemia-related adverse events (AEs) were more common with long-acting pasireotide, a SSA targeting a broader range of somatostatin receptor subtypes [9]. However, the mode of action of SSAs is complex as they have direct and indirect effects on glycemic control, regardless of GH and IGF-I [10, 11]. PEGV is the only GHR antagonist available; it is a competitive blocker that competes with endogenous GH for binding to the GHR [12–14]. Compared to SSA treatment, PEGV appears to be superior in improving glycemic control during the treatment of acromegaly patients [15, 16], and it has been shown to improve peripheral and hepatic insulin sensitivity in acromegaly [17, 18].

Established in 2004, ACROSTUDY™ is a global safety surveillance study of long-term PEGV treatment in acromegaly patients [19]. The objective of the present analysis was to explore the effects of PEGV on glucose metabolism parameters in patients enrolled in ACROSTUDY, according to their initial diabetes or non-diabetes status.

## Patients and methods

### Study protocol

ACROSTUDY is an ongoing open-label, international, non-interventional, post-marketing surveillance study open to patients with acromegaly who are treated with (or about to begin) PEGV. It is designed to monitor long-term safety and outcomes in routine clinical practice. Since this is a non-interventional study, treatment dosing regimen, as well as timing of follow-up visits, pituitary imaging, and laboratory evaluations are at the discretion of each treating physician/investigator [19].

The most important exclusion criteria are: participation in any clinical trial of an investigational drug for acromegaly, requirement for surgical decompression of the tumor (such as in contact with the optic chiasm) or non-medical therapy because of visual field loss, cranial nerve palsies, or intracranial hypertension. No specific additional diagnostic or monitoring procedures must be conducted as part of this study as previously reported [19]. The ACROSTUDY data reported here were collected in compliance with, and consistent with the most recent version of the Declaration of Helsinki. In addition, all applicable local laws and regulatory requirements in the countries involved were adhered to. Local ethical approval was obtained for all participating centers and all patients provided written informed consent prior to enrollment in the study.

### Diabetes and no-diabetes groups

We investigated parameters of glucose metabolism in patients followed in ACROSTUDY, using the database freeze performed on December 4th, 2012. Two cohorts, DM and no-diabetes (No-DM) were retrospectively identified. Presence of DM was determined on previous medical history, or on the basis of the following criteria: HbA1c ≥ 6.5%, or glucose >200 mg/dl as defined by American Diabetes Association (ADA) guidelines [20], or antidiabetic medication before PEGV start. IGT was defined using the same guidelines. Both cohorts were analyzed in a cross-sectional manner at yearly time points for 4 years and in a longitudinal manner 1 year and at least 4 years after PEGV start. For the longitudinal analysis, values obtained between 4 and 5 years were considered as 4-year data. Main demographic and anthropometric data, characteristics of PEGV administration, HbA1c, IGF-I at PEGV start, previous treatments for acromegaly, concomitant treatments for diabetes, and AEs were analyzed.

### Concomitant therapies

Treatment for diabetes was classified into 3 categories: category 1 included lifestyle measures only or in combination with metformin and/or acarbose; category 2 included any other oral antidiabetic agent or glucagon-like peptide 1 (GLP1) analogs; category 3 comprised any form of insulin treatment. Concomitant medications for acromegaly were evaluated to differentiate patients who were on PEGV only (monotherapy group) and those who received a combination (combination therapy group) of PEGV and SSAs and/or dopamine agonists (DA). For monotherapy, SSAs or DAs had to be stopped before or the same day as PEGV treatment was initiated for the baseline visit; at yearly visits, any SSA/DA treatment had to be stopped at least 4 months/2 months before the yearly IGF-I measurement. In the combination therapy group, at baseline, SSA and/or DA should be given continuously, or with a maximal gap of 6 weeks before the start of PEGV for long-acting SSA and 30 days for short-acting SSA and DA; at yearly visits, SSA and/or DA should be given continuously, or with a maximal gap of 6 weeks before the yearly IGF-I measurement for long-acting SSA and 30 days for short-acting SSA and DA.

### Laboratory tests and MRI evaluations

Hormonal and biochemical tests were realized at investigator’s discretion, using commercial assays available in each center, and interpreted according to the corresponding normal reference ranges. IGF-I data were reported in relation to local reference values. The IGF-I values were also expressed as percentage of the upper limit of age-adjusted normal values.

The protocol recommended that the local MRI procedures use the same imaging technique and equipment whenever possible throughout ACROSTUDY, namely T1 weighted spin-echo (or fast spin echo) sagittal and coronal images before and after gadolinium, and T2 weighted fast spin echo coronal images. If the local radiologist reported a significant change in pituitary tumor size, whether it was considered clinically important or not, all available images for that patient were to be considered for central assessment as previously described [19].

### Safety evaluations

Comorbidities including DM, hypertension, cardiovascular, and cerebrovascular diseases, respiratory tract disorders, osteoarthritis, benign, and malignant tumors, sleep apnea, and hepatic diseases, diagnosed before PEGV start were to be reported at study entry. Safety was evaluated by collection of AEs, serious adverse events (SAEs) and laboratory and MRI data as reported by investigators. For patients treated with PEGV before entering ACROSTUDY, AE collected prior to study entry were considered to be part of the medical history and reported in the database if deemed relevant. Any aggravation of a pre-existing condition during ACROSTUDY was to be reported as an AE. In addition to the analysis of new-onset DM reported as an AE, we also considered all patients who did not meet the criteria for diabetes as defined above at the start of the study but did so at any time point through the latest evaluation as developing DM and regarded them as a new onset DM.

### Statistical methods

All analyses were planned as descriptive summaries. The full analysis set consisted of all subjects who entered ACROSTUDY and received at least 1 dose of PEGV. Baseline was defined as the start of PEGV treatment, regardless of the date of enrollment into ACROSTUDY. All available data following PEGV start were to be summarized.

For testing differences in laboratory data between DM and no-DM patients, multiple regression analyses controlling for gender and age were used. The baseline test of age and BMI between the groups was performed using Wilcoxon rank test. For frequency tables Fischer’s exact test was used for 2 × 2 tables and a chi-square test otherwise. Correlation analyses were performed with Pearson correlation coefficients to analyze linear relationships between variations in IGF-I and variations in blood glucose. Mc Nemar’s test on paired nominal data was used to assess change between two-time points in the prevalence of patients with impaired glucose tolerance. *P* < 0.05 was considered significant. SAS® version 9.2 for Sun Solarix (SAS Institute, Cary, North Carolina), Proc GLM, Proc NPAR1WAY, Proc CORR, and Proc FREQ, was used for the different statistical analyses.

## Results

### Baseline characteristics and demographic data

Patients were included from 15 countries in Europe and North America (Table 1). A large majority (92.7%) was Caucasian. The overall study cohort consisted of 1762 patients (892 males), of whom 510 (28.9%) were included in the DM group and 1252 in the no-DM group. The diagnosis of DM was based on the ADA-derived definition of diabetes as an associated comorbidity at baseline in 485 patients, on an HbA1c value ≥6.5% in 11 patients, and on the presence of an antidiabetic drug among concomitant medications in 14 patients. There was a large variability in the reported rate of DM within countries, ranging from 16.7% (Great Britain) to 56.6% (Greece). Main patients’ clinical and demographic characteristics at baseline are summarized in Table 2. There was a significantly greater proportion of female patients in the DM group. Patients with DM were significantly older than those without DM at diagnosis of acromegaly, and this still held true at the time of PEGV start. As expected, patients with DM had a greater BMI than those without DM.

**Table 1:** Distribution of patients among participating countries

Country	Number of patients (%)
Germany	480 (27.2)
Italy	340 (19.3)
France	276 (15.7)
Spain	199 (11.3)
USA	120 (6.8)
Netherlands	108 (6.1)
Greece	53 (3.0)
Great Britain	48 (2.7)
Sweden	37 (2.1)
Denmark	32 (1.8)
Belgium	28 (1.6)
Slovakia	23 (1.3)
Portugal	9 (0.5)
Hungary	7 (0.4)
Austria	2 (0.1)

**Table 2:** Baseline characteristics and demographic data

Variable, unit	Diabetes (*n* = 510)	No-diabetes (*n* = 1,252)	*P*-value
Age at diagnosis, y	46.7 ± 13.45	39.7 ± 12.93	0.0001
Sex: M/F (%)	219/291 (43/57)	673/579 (54/46)	0.0001
Weight, kg	89.2 ± 19.12	87.4 ± 19.85	NA
Height, cm	168.7 ± 10.96	173.0 ± 11.70	NA
BMI, kg/m^2^	31.2 ± 6.00	28.6 ± 5.00	0.0001
Age at PEGV start, y	55.4 ± 13.47	49.1 ± 13.75	0.0001
Cause of GH hypersecretion (%)			
Microadenoma	40 (8)	61 (5)	
Macroadenoma	170 (34)	439 (36)	
Pituitary adenoma (not specified)	288 (57)	719 (59)	
Extrapituitary	1 (0.2)	3 (0.2)	
Not known	3 (0.6)	7 (0.6)	
Hypertension (%)			
Yes	347 (68.7)	472 (40.2)	0.0001
No	158 (31.2)	703 (59.8)	0.0001
CVD (%)			
Yes	364 (71.4)	513 (41)	0.0001
No	141 (27.7)	672 (53.7)	0.0001
Hyperlipidemia, *n* (%)	48 (9.4)	71 (5.6)	0.0045
IGF-I > ULN, n (% of patients with available samples)	281 (83.1)	672 (85.8)	NS

Data are mean ± SD or a proportion (%)

*CVD* cardiovascular disease (includes any cardiovascular comorbidity), *NS* not significant, *NA* not applicable

As shown in Table 3, most patients had received surgical and medical treatments for acromegaly before PEGV start, and only 16.3% had received medical treatment only.

**Table 3:** Treatments before pegvisomant start

	Diabetes (*n* = 510)	No-diabetes (*n* = 1,252)	All (*n* = 1,762)	*P*-value
Medical treatment only	103 (20.2%)	185 (14.8%)	288 (16.3%)	< 0.01
Surgery only	21 (4.1%)	50 (4.0%)	71 (4.0%)	NS
Radiotherapy only	0	1 (0.1%)	1 (0.1%)	NA
Medical treatment and surgery	212 (41.6%)	626 (50.0%)	838 (47.6%)	< 0.01
Medical treatment and radiotherapy	16 (3.1%)	24 (1.9%)	40 (2.3%)	NS
Surgery and radiotherapy	12 (2.4%)	21 (1.7%)	33 (1.9%)	NS
Medical treatment, surgery and radiotherapy	126 (24.7%)	293 (23.4%)	419 (23.8%)	NS

*NA* not available, *NS* not significant

The median duration of acromegaly before PEGV start was similar in DM and no-DM groups (4.7 and 4.1 years respectively). Mean duration of PEGV treatment was also similar in both groups (5.2 ± 2.7 yr vs. 5.4 ± 2.7 yr, respectively).

### Cross-sectional analysis

**Fig. 1:**
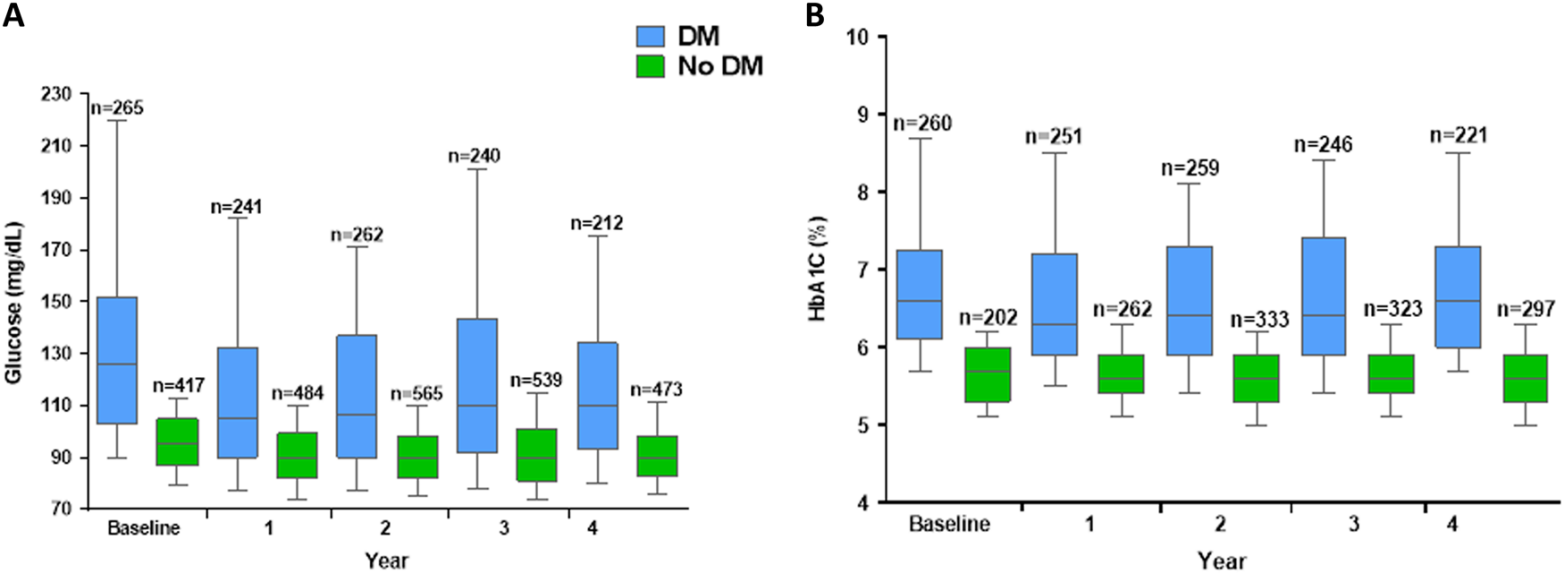
Cross-sectional analyses in patients with acromegaly treated with pegvisomant: Blood Glucose levels **a** and HbA1c **b** over time (yearly evaluations)

As shown on Fig. 1a, the cross-sectional analysis showed that fasting blood glucose values remained stable in the no-DM cohort throughout the follow-up period whereas in the DM group they decreased from 140.0 ± 57.8 mg/dl at baseline to 116.4 ± 44.8 mg/dl (*p* = 0.0001) at year 1, and 120.0 ± 44.3 mg/dl at year 4 (*p* = 0.0001) In parallel, as shown on Fig. 1b, HbA1c remained stable in the no-DM group. In the DM group, HbA1c was above 6.5% in 61.9% (161/260) of patients at baseline, 45.4% (114/251) at year 1 (*p* = 0.007) and 47.1–53.8% of patients at subsequent yearly time points over 4 years. Prevalence of IGT decreased from 11.2% at pegvisomant start to 8.0% at year 1, and 6.4% at year 4.

Between baseline and yearly evaluations, the proportion of patients with normalized IGF-I increased from 16.1% to 52.6–58.9% in the DM group, and from 12.4% to 60.8–62.0% in the no-DM group (NS).

In patients with increased IGF-I, PEGV dose was 16.4 ± 9.3 mg vs 14.7 ± 7.8 mg in DM vs non-DM groups, respectively at yr. 1, and 19.9 ± 11.4 mg vs 18.8 ± 9.3 mg at yr. 4 (for both, *p* = 0.00006).

### Longitudinal analyses

#### Analysis at 1 year

At year 1, in the patients for whom baseline and 1-year follow-up data were available, blood glucose changed from a mean of 132.8 ± 50.2 mg/dl to 116.5 ± 41.1 mg/dl in the DM group (*n* = 141) and from 95.7 ± 16.4 to 90.6 ± 17.1 mg/dl in the no-DM group (*n* = 210). The decrease was significantly more pronounced in the DM group (*p* < 0.0001). Similarly, the decrease in HbA1c values was significantly more marked in the DM group (*p* < 0.05), where it changed from a mean of 7.0 ± 1.5% to 6.6 ± 1.2% (*n* = 149) compared to a change from 5.7 ± 0.45% to 5.5 ± 0.45% in the no-DM group (*n* = 83).

In patients with elevated IGF-I levels at baseline, 52.1% (100/192) in the DM group, and 57.4% (283/493) in the no-DM group had normalized IGF-I levels at year 1, a difference that was not significant. However, to achieve normal IGF-I levels, DM patients had received a higher mean PEGV dose (18.2 mg/day vs. 15.3 mg/day for no-DM patients, *p* = 0.015).

Interestingly, we found that one year after pegvisomant start, there was a significant correlation between the delta IGF-I (*n* = 274) and delta glucose (*n* = 272), in the whole cohort, with a linear correlation coefficient *r* = 0.21 (*p* = 0.0008), as well as in both the DM and no-DM groups (*r* = 0.27; *p* = 0.007, and *r* = 0.20; *p* = 0.01, respectively).

#### Analysis at 4 years

**Fig. 2:**
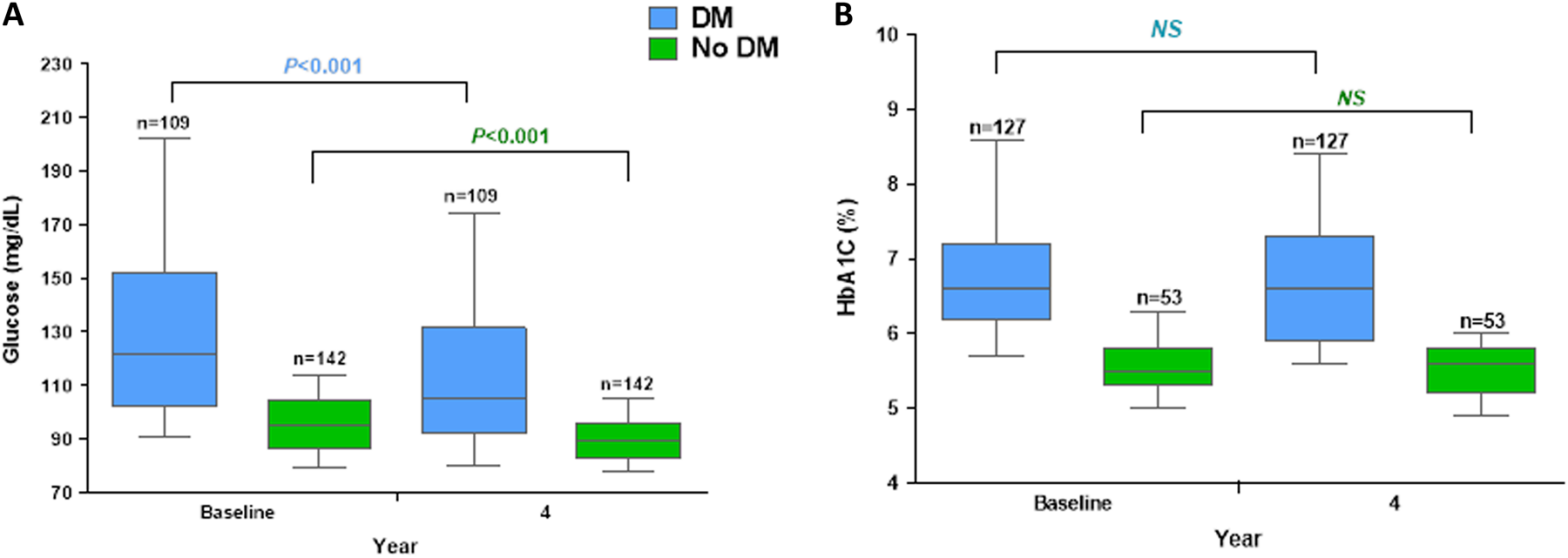
Longitudinal analyses in patients with acromegaly treated with pegvisomant: Blood Glucose levels **a** and HbA1c **b** over time (yearly evaluations)

Mean glucose levels changed from 136.3 ± 49.9 mg/dl at baseline to 116.1 ± 39.2 mg/dl at year 4 in the DM group (*n* = 109, mean decrease of 20.2 mg/dl) and from 94.3 ± 20.2 mg/dl to 90.5 ± 15.2 mg/dl in the no-DM group (*n* = 142, mean decrease of 3.9 mg/dl), (*p* < 0.0001) (Fig. 2a). In 127 DM patients with available values, HbA1c changed by 0.13 ± 1.5%, from 7.0 ± 1.4% to 6.8 ± 1.4% (NS) (Fig. 2b). HbA1c remained stable (data not shown) in the few no-DM patients in whom this value was determined. Among patients with elevated IGF-I at baseline, 53.0% (70/132) in the DM group, and 59.1% (201/340) in the no-DM group achieved normalization (NS). Four years after pegvisomant start, there was no significant correlation between the decrease in IGF-I and the change in glucose.

### Changes in diabetes treatment status

**Fig. 3:**
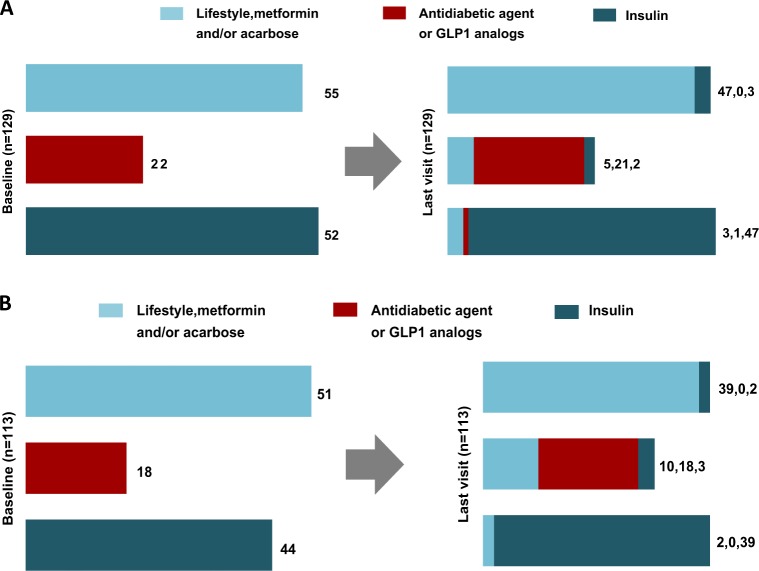
Change in DM treatment from PEGV start to last observation (6 months to 4 years) in the pegvisomant (PEGV) monotherapy group **a**, and in the combination group **b**: longitudinal analyses in 129 patients

Changes in DM treatment groups from PEGV start to last observation available (6 months to 4 years) are shown in Table 4 (cross-sectional analyses) and Fig. 3a (in the monotherapy group) and 3B (in the combination therapy group) for the longitudinal analyses. Overall, most of DM patients (87.2%) remained in the same group, while 4.1% and 8.7% changed to a less intensive or more intensive treatment category, respectively. Association of SSAs did not significantly affect treatment group allocation: in the monotherapy and the combination therapy groups, 89.1% and 85.0% remained in the same anti-diabetes treatment category while 7.0% and 10.6%, changed to a more intensive treatment group respectively.

**Table 4:** Cross-sectional analysis of antidiabetic treatments in patients with DM receiving pegvisomant monotherapy or combination therapy

	DM treatment category	Baseline *n* = 429	Year 1 *n* = 322	Year 4 *n* = 277
All Patients, *n* (%)	1	244 (57)	143 (44)	104 (37)
	2	49 (11)	70 (22)	66 (24)
	3	136 (32)	109 (34)	108 (39)
PEGV monotherapy	1	117 (60)	56 (43)	43 (36)
	2	23 (12)	31 (24)	28 (24)
	3	56 (28)	44 (36)	47 (40)
Combination therapy PEGV + SSA	1	79 (53)	58 (48)	38 (38)
	2	18 (12)	21 (17)	26 (26)
	3	51 (34)	41 (34)	35 (35)

1: Lifestyle intervention with or w/o metformin/acarbose, 2: Addition of any other kind of therapy except insulin, 3: Insulin with any other therapy

*PEGV* pegvisomant, *SSA* somatostatin analogues

### Safety evaluations

Table 5 summarizes all-cause and treatment-related AEs in the study population. The most common treatment-related AEs were general disorders and administration site conditions (1.8% in the DM cohort vs. 2.2% in the no-DM group), nervous system disorders (1.4% vs. 1.5%), gastrointestinal disorders (1.2% in the DM cohort), and skin and subcutaneous tissue disorders in the no-DM cohort (1.4%). Treatment-related liver test abnormalities were reported in 12 (2.4%) DM and in 50 (4.0%) no-DM patients. There were 18 (3.5%) and 24 (1.9%)—deaths (all-cause) in the DM and no-DM cohorts, respectively. None were considered treatment-related by the investigator. In the DM group, 36 patients (7.1%) withdrew study drug (temporarily or permanently) due to—SAEs (all-cause). In the no-DM group, drug withdrawal due to—SAEs (all-cause) occurred in 55 patients (4.4%).

**Table 5:** Summary of all-cause and treatment-emergent adverse events (AEs) and serious AE (SAEs)

	N	Patients with AEs, *n*	Treatment-emergent AEs, *n*	Patients with SAEs, *n*	Treatment-emergent SAEs, *n*
Diabetes	510	258	965 (95)	76	194 (12)
No-diabetes	1252	568	2094 (305)	125	368 (30)
Total	1762	826	3059 (400)	201	562 (42)

New onset DM was reported as an AE in 19 patients during the period of observation. At the time point when DM was reported as an AE, 8 patients were on pegvisomant monotherapy and 11 patients were on combination therapy.

Among patients with no DM at PEGV start, we identified—whether diabetes had been declared as an AE or not—those having at least one value of HbA1c and/or blood glucose above the cut-offs and/or start of an anti-diabetes drug during PEVG therapy. In total 82 fulfilled at least one of these criteria (49 HbA1c, 46 anti-diabetes drugs, 9 blood glucose values).

There was no reported case of hypoglycemia in the DM group and one case of hypoglycemia occurred in a 78-year old female patient from the non-DM group, 1 year after pegvisomant start.

## Discussion

We described here the effects of PEGV therapy on glucose metabolism in acromegaly patients enrolled in ACROSTUDY. This population mainly consists of patients in whom previous medical treatments failed to control acromegaly. Nevertheless, they seem to be representative of acromegaly patients described in several previous series, as regards age at diagnosis and characteristics of the disease [6, 21–25]. Previous ACROSTUDY reports have shown that undertreatment remains a major issue in daily clinical practice, highlighting the value of real-world data, and that inadequate control of acromegaly represents a risk factor for persistently altered glucose metabolism (19). This is confirmed by our present results showing that, one year after pegvisomant start, there was a significant correlation between delta IGF-I and delta glucose, in both DM and no-DM groups. This suggests that correction of IGF-1 excess by antagonizing GH-receptor activity (rather than a direct effect of the drug) is likely to account for the outcome of glucose parameters in treated patients.

### Prevalence of diabetes

We observed in our cohort that the prevalence of diabetes was very different among countries. This finding is consistent with data from national registries [21–25]. In the German Pegvisomant Observational Study (GPOS), it was 37.6%, compared to 6.9% in a control group, representative of the general population [26]. Interestingly, the Liege Acromegaly Survey, based on 3173 patients [6], noted a significant relationship between glucose levels and IGF-1 concentrations. DM has indeed been shown to be correlated with high IGF-I levels [6, 20, 26, 27], but not with GH levels [21, 28, 29]. We did not observe this finding in our study but it was noted that, as in many other series, DM was associated with older age [21, 22, 25, 27, 28], and higher BMI [25, 27, 28]. Yet, DM carries overall poorer prognosis, with increased mortality in acromegaly patients [22, 30].

### Effects of treatments on glucose metabolism

Impaired glucose metabolism in acromegaly is mainly due to insulin resistance, resulting from chronic excessive GH exposure. Therefore, any treatment that normalizes GH/IGF-I should improve insulin sensitivity and decrease glucose intolerance, provided that the treatment does not also decrease insulin secretion as well, as is the case with SSAs. Therefore, the results with SSAs are more nuanced: although they can improve glucose parameters by reducing GH/IGF-I concentrations, they may also impair glucose tolerance because of complex interactions between somatostatin receptors and glucose regulatory hormones, and inhibition of beta cells secretory functions [7, 31]. The benefits of treatment on glucose metabolism have been clearly demonstrated for surgery [32].

In a study by Colao et al., 100 patients treated with surgery only, SSAs only or both presented with deterioration of glucose tolerance after 60 months [33]. Another study on 45 de novo patients with a 5-year first-line treatment with SSA concluded that the prevalence of IGT and DM did not change significantly during this period [34]. Glucose metabolism was also investigated in 51 patients, among which 18 were treated with lanreotide autogel and 33 had no medical treatment [35]. Differences were shown between groups: HOMA-R was similar in both but HOMA-β was significantly lower in treated patients, suggesting that SSAs decrease β-cell function but do not affect insulin resistance [35]. The multireceptor-targeted SSA pasireotide was found in healthy volunteers to increase glucose levels, while hepatic and peripheral insulin sensitivity remained unchanged [36]. In medically naïve patients with acromegaly, pasireotide was compared to octreotide in a prospective randomized double-blind study, and resulted in higher rates of hyperglycemia-related events (57.3% vs 21.7%) [9].

In contrast, glucose metabolism improves on PEGV, as shown in our series, with a significant decrease in mean glucose levels in DM patients while they remained stable in no-DM patients. We also observed a significant decrease in the proportion of patients with IGT from 11.2% at pegvisomant start to 6.4% at year 4. However, in the context of an observational study not designed to address any efficacy endpoint—and due to a substantial proportion of missing data especially on glucose metabolism—the decrease in HbA1c was not statistically significant. These effects are probably mediated by an increased insulin sensitivity, which in turn lowers glucose levels and reduces beta-cell stimulation, and eventually restores insulin response to glucose exposure [37], as shown by previous studies [17]. This is unlikely to be attributable to a direct antidiabetic effect of the drug but may rather be due to an indirect effect due to the decreased activation of GH receptors secondarily resulting in improved insulin sensitivity. Similarly, Barkan et al showed that patients switched from octreotide LAR to PEGV had decreased glucose and HbA1c levels after 32 weeks, whether patients were diabetic or not at the start of treatment [15]. In a study with sequential treatment (SSA, followed by no treatment, then PEGV then SSA + PEGV), performed in 11 patients refractory to SSA alone [37], glucose levels were the lowest during PEGV therapy and the highest during SSA treatment, intermediate values being reported during combined treatment.

In the first large cohort of patients published in 2001 by Van der Lely et al., patients had a decrease in fasting insulin and fasting glucose while HbA1c did not change significantly [38]. In the German ACROSTUDY cohort [39], the mean duration of PEGV was 174 weeks, 70% of patients received monotherapy while the remaining 30% had combined treatment (PEGV + SSA). In the whole group of DM patients with available data, the decrease in HbA1c was statistically significant but a sub-group analysis showed that it remained significant only in the monotherapy group although the IGF-I response was of the same order of magnitude. In our cohort, almost 90% of DM patients remained in the same antidiabetic treatment category. We could not find differences between those receiving PEGV monotherapy and those with combined treatment (PEGV + SSA). However, these data were collected at different time points, and in a limited number of patients, which is probably insufficient to show differences if any. Of note, the present study was not designed to compare the respective effects of PEGV and SSA alone or in combination on glucose metabolism.

### Other treatment effects

In our cohort, the efficacy of PEGV appeared similar in DM and no-DM patients, since the proportions of those who normalized IGF-I were the same in both groups. However, in patients with normal IGF-I levels after 1 year, the mean PEGV dose was higher in DM than in no-DM patients (18.2 mg/d vs 15.3 mg/d, *p* = 0.015). In the German ACROSTUDY cohort, patients with DM achieved IGF-I normalization less frequently than non-diabetic ones, but they also required higher PEGV doses to normalize IGF-I (18.9 vs 15.5 mg/day, *p* < 0.01). This difference was even more pronounced in patients treated with insulin (22.8 vs 17.2 mg/day for those treated with oral antidiabetic drugs, *p* = 0.11) [39]. This is in line with the concept that low insulin levels in the portal circulation reduce IGF-I generation by the liver, while high portal insulin concentrations increase the liver sensitivity for GH by increasing the number of GHR at the hepatocytes’ surface [40]. The vast majority of our DM patients had type 2 diabetes, thus probably hyperinsulinemia and then, more GH receptors in the liver [40]. Since PEGV is a competitive blocker, there is a need for more drug to block GHRs when their number increases, which probably explains the observed need for a higher dose in the controlled DM patients than in the no-DM group.

### Limitations

This is a large observational/non-interventional study; however, some parameters were not analyzed adequately, due to missing data. Moreover, ACROSTUDY is not designed to evaluate specifically glucose metabolism issues and investigators may have focused more on acromegaly data than on comorbidities, glucose tests, diabetes medications, or HbA1c. For this reason, some analyses are restricted to a small percentage of the initial cohort. Furthermore, the study did not allow a strict analysis of the role of IGF-I control on the outcome of glucose metabolism. Thus, the actual data are not sufficient to distinguish between a possible effect of pegvisomant on glucose metabolism and an indirect effect through the normalization of IGF-I. Likewise, we did not investigate the mechanisms of glucose parameters changes by measuring insulin sensitivity of beta-cell functions. Therefore, we did not perform additional analyses to differentiate between patients receiving PEGV monotherapy or combination therapy.

## Conclusion

In this observational, international, surveillance study, we demonstrated that PEGV can be used effectively and safely in diabetes patients with acromegaly. The treatment resulted in a moderate decrease in mean glucose levels. The efficacy was similar in diabetes and non-diabetes patients. The safety profile was similar in both groups, without any unexpected adverse event. Due to these effects on glucose metabolism, PEGV appears to be a favorable therapeutic option in diabetes patients with acromegaly.
